# Efficiency of Different Endodontic Irrigation and Activation Systems in Removal of the Smear Layer: A Scanning Electron Microscopy Study

**DOI:** 10.22037/iej.v12i4.9571

**Published:** 2017

**Authors:** Priyatam Karade, Rutuja Chopade, Suvarna Patil, Upendra Hoshing, Madhukar Rao, Neha Rane, Aditi Chopade, Anish Kulkarni

**Affiliations:** a *Department of Conservative Dentistry and Endodontics, Vasantdada Patil Dental College and Hospital, Kavalapur, Sangli, India; *; b *Bharati Deemed University Dental College and Hospital, Sangli, India; *; c *Department of Conservative Dentistry and Endodontics, MGV’s KBH Dental College and Hospital, Panchwati, Nashik, India; *; d *Private Practioner, Kolhapur, India; *; e *Private Practioner, Pune, India*

**Keywords:** EndoVac Irrigation System, Passive Ultrasonic Irrigation, Smear Layer, Sonic Irrigation System

## Abstract

**Introduction:**

This *in vitro* study was designed to evaluate and compare different endodontic irrigation and activation systems for removal of the intracanal smear layer.

**Methods and Materials:**

Forty recently extracted, non-carious human intact single rooted premolars were selected and divided into five groups (*n*=10) according to the root canal irrigation systems; syringe and needle irrigation (CTR), sonic irrigation, passive ultrasonic irrigation (PUI) and EndoVac irrigation system. All groups were prepared to #40 apical size with K-files. Each sample was subjected to final irrigation by using four different irrigation/activation systems. After splitting the samples, one half of each root was selected for examination under scanning electron microscope (SEM). The irrigation systems were compared using the Fisher's exact test with the level of significance set at 0.05.

**Results:**

The four groups did not differ from each other in the coronal and mid-root parts of the canal. In the apical part of the canal none of the methods could completely remove all the smear layer but EndoVac system showed significantly better removal of smear layer and debris than the other methods.

**Conclusion:**

Within the limitations of the present study, the EndoVac system cleaned the apical part of the canal more efficiently than sonic, ultrasonic and syringe and needle irrigation.

## Introduction

The ultimate goal of endodontic therapy is to bring the involved teeth to a state of health and function. Cleaning and shaping of the root canal system is recognized as being one of the most important stages in root canal treatment [1].

Irrigants can augment mechanical debridement by flushing out debris, dissolving tissue, and disinfecting the root canal system [2]. An effective irrigation delivery system is required for the irrigants to reach the working length. Such a delivery system should have adequate flow and deliver sufficient volume of irrigant all the way to working length to be effective in debriding the complete canal system [1].

Root canal irrigation systems can be divided into two broad categories, manual delivery and agitation techniques and machine-assisted agitation devices [3]. Manual irrigation includes positive pressure irrigation, commonly performed with a syringe and a side-vented needle [4]. On the other hand, machine-assisted irrigation techniques include sonic and ultrasonic as well as newer systems like apical negative pressure irrigation and the plastic rotary file [5, 6].The apical part of the canal, with its cul-de-sac configuration, presents a special challenge and several studies have indicated that syringe and needle irrigation tends to leave this parts of the canal covered with smear layer and debris, despite application of ethylenediaminetetraaceticacid (EDTA) [7].

Tronstad was the first to report the use of a sonic instruments for endodontic purposes in 1985 [8]. Sonic irrigation is different from ultra-sonic irrigation in that it operates at a lower frequency (1-6 kHz) and produces smaller shear stresses. The sonic energy also generates significantly higher amplitude or greater back-and-forth tip movement [9]. Ultrasonic devices had long been used in periodontics before Richman introduced ultrasound to endodontics as a means of canal debridement in 1957. Compared with sonic energy, ultrasonic energy produces high frequencies with low amplitudes. The files are designed to oscillate at ultrasonic frequencies of 25-30 kHz [9]. 

EndoVac (Discus Dental, Culver City, CA, USA) represents a novel approach to irrigationas, instead of delivering the irrigant through the needle, the EndoVac system is based on a negative-pressure approach whereby the irrigant placed in the pulp chamber is sucked down the root canal and back up again through a thin needle with a special design [10]. 

To the best of our knowledge, there are very limited data in the literature comparing the root canal irrigant agitation systems for smear layer removal. Hence, the aim of this *in vitro* study is to evaluate and compare four different endodontic irrigation systems including conventional needle irrigation, sonic, ultrasonic and EndoVac irrigation in efficacy of intracanal smear layer removal using scanning electron microscopy (SEM).

## Materials and Methods

A total of forty recently extracted, non carious single rooted human intact premolars were selected. Endodontic access was obtained with round diamond bur and #15 K-file (Dentsply Maillefer, Ballaigues, Switzerland) was introduced into the root canal until the tip was just visible at the apical foramen. Working lengths were set by deducting 1 mm from lengths of the files when they were extruded just beyond the apical foramina. Crowns were sectioned using diamond disc to obtain a standard working length of 16 mm for all samples. To simulate clinical conditions, apices were sealed with hot glue.

Forty teeth were subjected to manual root canal instrumentation, using the step-back method. The root canals were first instrumented manually with K-files up to # 40 master apical size, along with irrigation with 5 mL of 5.25% NaOCl after each instrument. The step back phase of the apical third began with the # 45 K-file and 5 sequentially larger K-files up to #70. These roots were then randomly divided into 4 groups (*n*=10). Then each sample was subjected to final irrigation by using four different irrigation systems with 5 mL 5.25% NaOCl, followed by 5 mL of 17% EDTA, followed by 5 mL 5.25% NaOCl and 0.9% normal saline [11].

***Grouping***

***Final syringe and needle irrigation*** ***: ***Final irrigation was done with 5 mL 5.25% NaOCl, followed by 5 mL of 17% EDTA, followed by 5 mL 5.25% NaOCl. Irrigation was done using syringe (Unolock, Hindustan syringes, Faridabad, India) adapted with 26 gauge monojet endodontic irrigation needle (Tyco Healthcare, Gosport, UK); no activation was applied in this group, which served as control [12].

***Final irrigation with sonic activation***: Final irrigation was conducted with sonic activation of the irrigants, using the sonic MM1500 handpiece system (Micromega, Besançon, France), adapted with #15/0.02 sonic file. The final irrigation consisted of 5 mL of 5.25 % NaOCl with 1 min of sonic activation. This was followed by 5 mL 17% EDTA, with 1 min activation and then by 5 mL of 5.25% NaOCl which was also activated for 1 min. The tip of the sonic file was applied at 1 mm short of the working length [13].

***Final irrigation with ultrasonic activation*** **: **Final irrigation was conducted with passive ultrasonic activation of the irrigants, using Minipiezon ultrasonic irrigation system (EMS, Nyon, Switzerland), adapted with a #20 Irrisafe ultrasonic files (Satelec, Acteon, Merignac, France). The ultrasonic file was placed into the canal 1 mm short of the working length without touching the walls and was activated at power setting of 4.

The final irrigation consisted of 5 mL of 5.25 % NaOCl with 1 min of activation. This was followed by 5 mL 17%EDTA, with 1 min activation and then by 5 mL of 5.25% NaOCl which was also activated for 1 min [11].

***Final irrigation with the EndoVac system: ***Final irrigation was conducted with the EndoVac (Axis-SybronEndo, Coppell, TX, USA) which was used according to manufacturer's instructions. The procedure consisted of 4 cycles of irrigation, each beginning with 30 sec of vacuum assisted irrigation followed by 30 sec of "soaking" (leaving the solution in the canal with no action). The first cycle was done using the macrocannula which was inserted to 1 mm from working length while the three following cycles were performed with the microcannula which was inserted to 9 mm from working length. In the first and second cycles 5.25% NaOCl was used. In the third cycle 17% EDTA was used which was followed by the forth cycle in which 5.25% NaOCl was used again [14].

At the end all groups were irrigated with 5 mL 0.9% normal saline and dried with absorbent paper points.

***Splitting the samples***

Deep grooves were made on the buccal and palatal surfaces of the roots, using diamond discs, without perforating into the canal. The roots were then split longitudinally using a chisel. One half of each root was selected for examination under SEM [11].

***Scanning electron microscope*** ***evaluation***

After assembly on coded stubs, the specimens were gold sputtered (JEOL, JFC*-*1600 Auto Fine Coater*,* Tokyo, Japan) and examined under 1000× magnification (JEOL*,* JSM*-*7600F*, *Tokyo, Japan). The dentinal wall of the coronal, middle and apical thirds was observed for the presence/absence of smear layer and visualization of the entrance to the dentinal tubules and representing photomicrographs were taken.

The images were examined and scored according to the criteria given by Hulsmann in 1997 [7]: *A.* All dentinal tubules were open and no smear layer was present, *B.* Some dentinal tubules were open and the rest covered by a thin smear layer, *C.* Few tubules were open and the rest covered by a thin homogeneous smear layer, *D.* All tubules were covered by a homogenous smear layer without any open tubule visible and *E.* A thick homogenous smear layer completely covered the canal walls.

Scoring was done by three independent examiners who were blinded to the group each specimen belonged. Inter-examiner agreement was 95% for the smear layer removal (according to Kappa test).When disagreement occurred as to the score of a given specimen (rarely), the issue was discussed to reach an agreement.

***Statistical analysis***

The four groups were compared to each other at the coronal, mid-root and apical part of the canal. Fisher’s exact test for nonparametric values was used for this comparison with significance set at 0.05. For purpose of this analysis the scores were grouped in two groups (Table 1): “clean or almost clean” which included scores “A” and “B” and “covered with smear layer” which included scores “C”, “D” and “E”.

## Results

**Figure 1:**
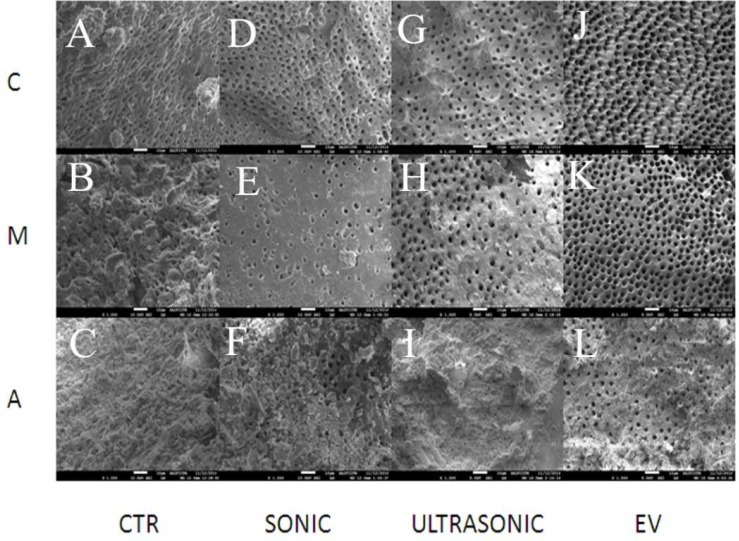
*Re* *presentative scanning electron microscopic* *samples of each group* *(original magnification 1000×): *A)* Syringe and needle irrigation at coronal third level; *B)* Syringe and needle irrigation at mid-root level; *C)* Syringe and needle irrigation at apical third level; *D)* Sonic irrigation at coronal third level; *E)* Sonic irrigation at mid-root level; *F)* Sonic irrigation at apical third level; *G)* PUI at coronal third level; *H)* PUI at mid-root level; *I)* PUI at apical third level; *J)* EndoVac at coronal third level; *K)* EndoVac at mid-root level; *L)* EndoVac at apical third level*

The results of the SEM evaluation are presented in table 1. In the coronal part there was no difference among the groups (Figure 1). In the mid-root section the results of the PUI and EndoVac groups tended to be better than syringe and needle and sonic activation groups, but the difference was not significant. 

At apical third region, none of the groups presented with dentin surface totally devoid of smear layer (Score “A”, Table 1) but in the EndoVac group the dentin surface at the apical part of the canal were cleaner and presented with “clean and almost clean” score in 60% and 80% of the cases, respectively which differed significantly from the other groups (*P*=0.011 and *P*=0.001, respectively). 

**Table 1:** Evaluation of canal walls by scanning electron microscopy (*Number of samples presenting with a given score

	**Score**	**Grouped**	**Syringe and Needle**		**Sonic irrigation**		**PUI**		**EndoVac**	
**Coronal**	A	Clean and Almost Clean	0 *	90%	0	90%	2	100%	1	100%
	B		9		9		8		9	
	C	Covered with Smear Layer	1	10%	1	10%	0	0%	0	0%
	D		0		0		0		0	
	E		0		0		0		0	
**Midroot**	A	Clean and Almost Clean	0	70%	0	80%	0	90%	1	90%
	B		7		8		9		8	
	C	Covered with Smear Layer	3	30%	2	20%	1	10%	1	10%
	D		0		0		0		0	
	E		0		0		0		0	
**Apical**	A	Clean and Almost Clean	0	0%	0	0%	0	0%	0	60%
	B		0		0		0		6	
	C	Covered with Smear Layer	0	100%	7	100%	9	100%	4	40%
	D		8		3		1		0	
	E		2		0		0		0	

## Discussion

It is important that the irrigants must be brought into direct contact with the entire canal wall surfaces for effective action particularly in the apical portions of root canals because of the typically challenging complexity of the root canal morphology. For the irrigants to reach the apical region there must be an effective delivery system. Various irrigation delivery and agitation systems have been developed for effective root canal irrigation [1].

For EndoVac group the effectiveness of EndoVac system in producing clean canals might be attributed to its apical negative pressure approach [15].The apical negative pressure pulls the irrigant down the canal walls towards the apex, creating a rapid turbulent current force towards the terminus of the microcannula. The orifices of the microcannula evacuate debris from the closed end of the canal systems. This mechanism helps to overcome the vapor lock, thus enabling effective irrigation [15].

Our results are consistent with the findings of Ribeiro *et al. *[16] who reported EndoVac to remove significantly more debris than NaviTip. Saber and Hashem [17] in their study also found that EndoVac was significantly better in removing debris than NaviTip in the apical third of the root canal. 

Passive ultrasonic irrigation (PUI) produced better cleaner canals than passive sonic irrigation [18]. This has been attributed to acoustic streaming and cavitation produced by the ultrasonically activated file [19-21]. The Sonic Air Micro-Mega handpiece with a Rispi-Sonic file was originally developed for shaping of the root canals. When used as an adjunct to irrigation, it was shown to remove debris more efficiently than needle irrigation but was no better than PUI [13, 22]. Sabins *et al.* [13] and Capar *et al.*[23] reported that passive ultrasonic irrigation produced significantly cleaner canals than passive sonic irrigation. However, Rodig *et al. *[24] Showed significantly greater smear layer removal when the Endo Activator was used rather than ultrasonic agitation and a canal brush.

In the present study the conventional syringe and needle irrigation system showed larger amount of debris and smear layer at apical, middle and coronal level than any other system because flushing action of syringe irrigation is relatively weak and is dependent not only on the anatomy of the root canal but also on the depth of placement and the diameter of the needle. It has been shown that irrigants can only progress 1 mm beyond the tip of the needle [18].

## Conclusion

Within the limitations of the present *in vitro* study, it can be concluded that none of the techniques completely removed all the smear layer from root canal walls at the apical part of the canal. Nevertheless, EndoVac system showed significantly better cleaning than syringe and needle, sonic and passive ultrasonic irrigation systems.
